# Detection of heteroplasmy and nuclear mitochondrial pseudogenes in the Japanese spiny lobster *Panulirus japonicus*

**DOI:** 10.1038/s41598-021-01346-8

**Published:** 2021-11-05

**Authors:** Seinen Chow, Takashi Yanagimoto, Haruko Takeyama

**Affiliations:** 1 Research Organization for Nano and Life Innovation, Waseda University, 513 Wasedatsurumaki-cho, Shinjuku-ku, Tokyo, 162–0041 Japan; 2 Fisheries Resources Institute, Japan Fisheries Research and Education Agency, Fukuura 2-12-4, Yokohama, Kanagawa 236-8648 Japan; 3 Department of Life Science and Medical Bioscience, Waseda University, 2-2 Wakamatsu cho, Shinjuku, Tokyo 162-8480 Japan; 4 Computational Bio Big-Data Open Innovation Laboratory, AIST-Waseda University, 3-4-1 Okubo, Shinjuku-ku, Tokyo, 169–0072 Japan

**Keywords:** Evolutionary genetics, Molecular evolution, Ecology, Evolution, Molecular biology, Zoology

## Abstract

Partial mtDNA cytochrome oxidase subunit I (COI) fragments and near entire stretch of 12S rDNA (12S) and control region (Dloop) of the Japanese spiny lobster (*Panulirus japonicus*) (n = 3) were amplified by PCR and used for direct nucleotide sequencing and for clone library-based nucleotide sequence analysis. Nucleotide sequences of a total of 75 clones in COI, 77 in 12S and 92 in Dloop were determined. Haplotypes of the clones matched with those obtained by direct sequencing were determined to be genuine mtDNA sequence of the individual. Phylogenetic analysis revealed several distinct groups of haplotypes in all three regions. Genuine mtDNA sequences were observed to form a group with their closely related variables, and most of these variables may be due to amplification error but a few to be heteroplasmy. Haplotypes determined as nuclear mitochondrial pseudogenes (NUMTs) formed distinct groups. Nucleotide sequence divergence (K2P distance) between genuine haplotypes and NUMTs were substantial (7.169–23.880% for COI, 1.336–23.434% for 12S, and 7.897–71.862% for Dloop). These values were comparable to or smaller than those between species of the genus *Panulirus*, indicating that integration of mtDNA into the nuclear genome is a continuous and dynamic process throughout pre- and post-speciation events. Double peaks in electropherograms obtained by direct nucleotide sequencing were attributed to common nucleotides shared by multiple NUMTs. Information on the heteroplasmy and NUMTs would be very important for addressing their impact on direct nucleotide sequencing and for quality control of nucleotide sequences obtained.

## Introduction

Mitochondrial DNA (mtDNA) has been widely used in molecular phylogenetics, population genetics, and DNA barcoding in animals due to its rapid evolutionary rate, little recombination, strict maternal inheritance, and homoplasmy within individual. On the other hand, there have been increasing number of reports on nuclear mitochondrial pseudogene referred to as “NUMT”^1,2^ and heteroplasmy^3,4^ in a wide range of eukaryotes. Direct nucleotide sequencing for PCR amplicons of mtDNA has been a conventional tool to detect sequence variation within and between species, however, electropherograms with double peaks or that are completely unreadable are sometimes encountered. These may be attributed to contamination of non-specific amplicons or to point mutations and length variation in heteroplasmic copies or NUMTs. Since stronger signals of the double peaks in electropherogram are preferentially adopted and unreadable electropherograms are discarded as “sequencing failed”, the incidences of heteroplasmy and NUMTs are likely to be underestimated. Since the number of heteroplasmic copies and NUMTs may be much smaller than that of the genuine mtDNA molecules, one might expect that heteroplasmy and NUMTs have little negative impact on the quality of nucleotide sequences obtained by direct nucleotide sequencing. However, consistent problems with obtaining good electropherograms in PCR-amplified mtDNA cytochrome oxidase subunit I (COI) fragments have been reported in some crustacean species^5–7^, in which multiple DNA sequences similar to a COI gene were detected from a single individual. In addition to these technical issues, unnoticed incorporation of heteroplasmic copies and NUMTs may lead to overestimation of population diversity and the number of species^2,5,8^.

We have frequently had difficulty for obtaining good electropherograms produced by direct nucleotide sequencing for COI, 12S rDNA (12S) and control region (Dloop) of the Japanese spiny lobster (*Panulirus japonicus*) and hypothesized that heteroplasmy was the major suspect for the issue. Then, we first begun to investigate extent of heteroplasmy in these regions but eventually found a fair number of NUMTs. To the best of our knowledge, this is the first investigation for the extent of heteroplasmy and NUMTs in the Japanese spiny lobster as well as their impact for direct nucleotide sequencing.

## Results

### Direct nucleotide sequencing

Readable electropherograms were obtained from both direction in COI fragments of all three individuals of the Japanese spiny lobster. COI sequences determined by direct nucleotide sequencing ranged from 807 to 864 bp and have been deposited in International Nucleotide Sequence Database Collection (INSDC) under accession numbers of LC571524‒LC571526. No stop codon was observed in these sequences (designated by PJK1-direct, PJK2-direct, and PJK3-direct). No indel was observed between these sequences. All nucleotide substitutions at 19 variable sites observed between these sequences were transition at the 3rd position of a codon, and all substitutions were synonymous. The mean Kimura two parameter (K2P) distance between these three haplotypes was 1.510 ± 0.352% SE and that between these sequences and a reference sequence of *P. japonicus* (NC_004251) was 1.087 ± 0.270%, which were all well within the range reported for Japanese spiny lobster samples collected in Japan and Taiwan^9,10^.

Electropherograms obtained by forward primer for 12S fragments were not readable, while those by reverse primer were readable in all individuals. 12S sequences determined by direct nucleotide sequencing using reverse primer alone ranged from 551 to 570 bp and have been deposited in INSDC under accession numbers of LC605705‒LC605707. Of nine variable sites, eight were transition and one was indel. The mean K2P distance between these three haplotypes (designated by PJK1-12Sdirect, PJK2-12Sdirect, and PJK3-12Sdirect) was 0.970 ± 0.338%, and that between these sequences and a reference sequence of *P. japonicus* was 0.835 ± 0.282%.

Electropherograms obtained by both primers for Dloop fragments were readable only in one individual (PJK2). This Dloop sequence determined by direct nucleotide sequencing was 762 bp and deposited in INSDC under accession number of LC605749. K2P distance between this haplotype (designated by PJK2-Dloopdirect) and a reference sequence of *P. japonicus* was 3.666%. No indel was observed between the two sequences, and 25 of 27 variable sites were transition.

### Phylogenetic analysis of clones, heteroplasmy and NUMTs

Among the 36–42 positive COI clones examined per individual, sequences (809–892 bp) of 22–31 clones per individual (75 clones in total) were successfully determined. After alignment, both ends of all sequences were trimmed to fit the shortest sequence obtained by direct nucleotide sequencing, yielding 774–810 bp sequences. Eleven clones of PJK1 were identical to PJK1-direct, as well as seven of PJK2 to PJK2-direct and three of PJK3 to PJK3-direct. These dominant haplotypes (807 bp) were determined to be genuine COI haplotypes of each individual, and representative sequences of these three genuine haplotypes were deposited in INSDC (LC 571527, LC571533 and LC571538). Nucleotide sequences of the remaining 54 clones were all different one another, in which 20 haplotypes were observed in PJK1, 14 in PJK2, and 20 in PJK3 (LC571541–LC571577, OK429332–OK429343, LC654683-LC654687).

**Figure 1:**
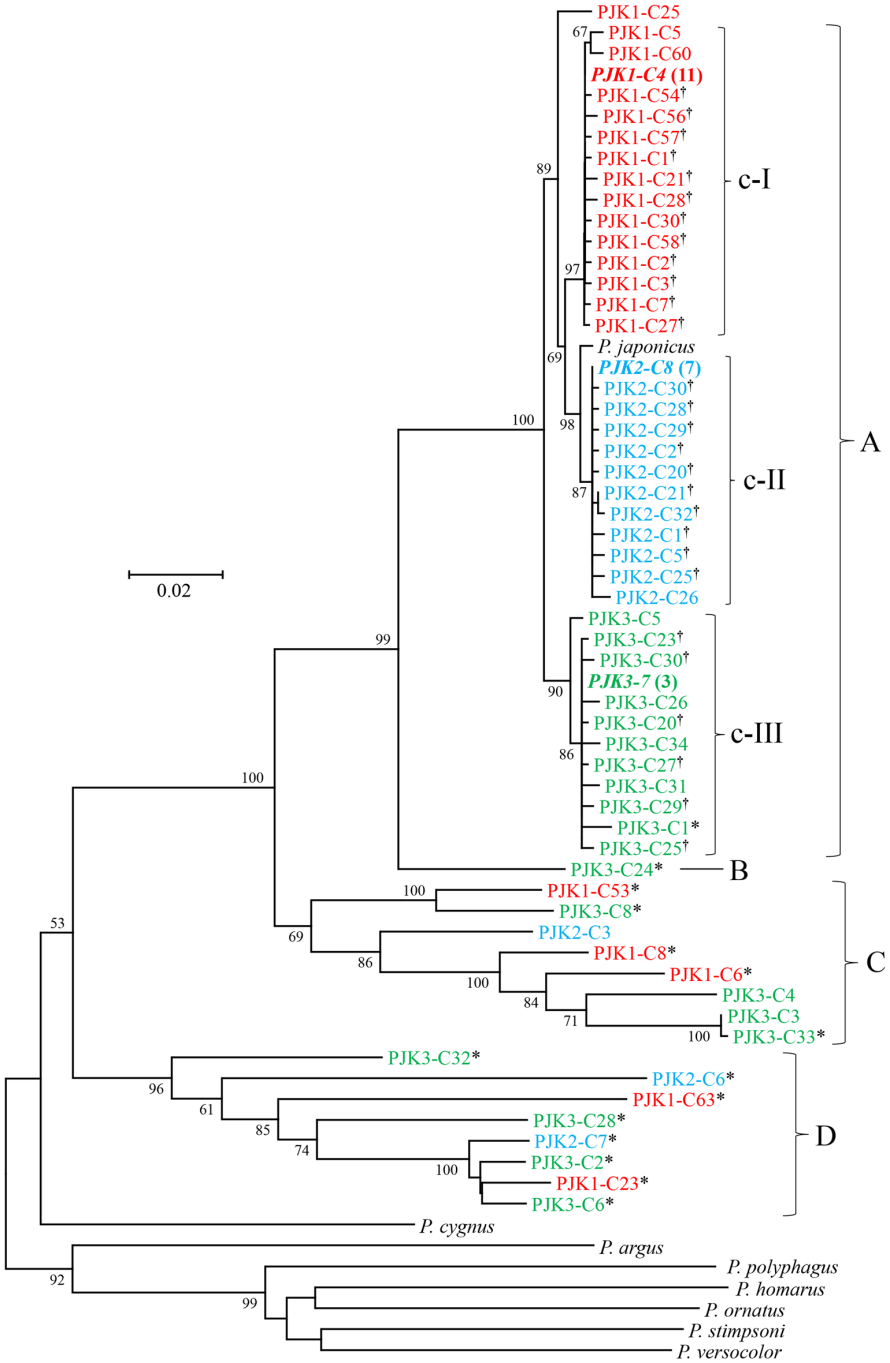
Neighbor-joining phylogenetic (NJ) tree showing relationships among 57 different haplotypes of cytochrome oxidase subunit I (COI) or COI-like sequences obtained from the Japanese spiny lobster (*Panulirus japonicus*), and COI sequences of eight congeneric species derived from the GenBank database. Haplotypes detected from the same individual of the Japanese spiny lobster share the same color. Genuine mtDNA haplotype is shown in bold italic and number of clones examined is shown in parenthesis. Stop codons were observed in haplotypes carrying asterisk. Haplotypes carrying dagger differ from the corresponding genuine mtDNA haplotype by less than two nucleotides (including indel). The bootstrap values greater than 60% (out of 1000 replicates) are shown at the nodes.

Phylogenetic tree constructed using three genuine COI haplotypes, 57 unique haplotypes and eight sequences of reference lobster species is shown in Fig. 1. Haplotypes detected from *P. japonicus* were segregated into four groups (designated by A, B, C and D). Among the outgroup species used, Australian rock lobster (*P. cygnus*) that morphologically and genetically belongs to the *P. japonicus* group^11,12^, appeared to be the closest kin to all haplotypes detected from *P. japonicus*. All haplotypes in group A were of the same length (807 bp), and no indel was observed. Three distinct clades (designated by c-I to c-III) were observed in group A, in which 14 haplotypes from PJK1, 11 from PJK2 and 11 from PJK3 were cohesively clustered together with their corresponding genuine haplotypes (bold italic). PJK1-C25 was outlier, having 10 nucleotide differences from the genuine COI sequence. The numbers of variable nucleotide sites between haplotypes within c-I, c-II and c-III were 20, 15 and 26, respectively, of which nonsynonymous nucleotide substitutions were observed at 11, 13 and 10 sites. Stop codon was observed only in one haplotype (PJK3-C1). The mean K2P distance between different haplotypes within these clades ranged from 0.320 ± 0.075 to 0.561 ± 0.103%. The mean K2P distances between three clades ranged from 1.343 ± 0.339 to 2.178 ± 0.464%. Although group A must be composed of sequences containing those caused by *Taq* polymerase error or true heteroplasmic sequences as well as genuine haplotypes, it is difficult to determine the former two categories. All of the non-genuine haplotypes in group A had singleton difference one another, supporting the occurrence of *Taq* polymerase error. We determined haplotypes (marked with dagger in Fig. 1) differed by less than two substitutions from the genuine haplotype to be due to *Taq* polymerase error. This criterion may be reasonable, since *Taq* polymerase-mediated errors were estimated to occur approximately at a frequency of 7.2 × 10^−5^ per bp per cycle^13^ to one mutation per 10,000 nucleotides per cycle^14^. When *Taq* polymerase error is taken into account, these K2P distances within and between clades and number of haplotypes are likely to be somewhat overestimated. PJK1-C25, two (PJK1-C5 and PJK1-C60) in c-I clade, one (PJK2-C26) in c-II, and five (PJK3-C1, PJK3-C5, PJK3-C26, PJK3-C31, PJK3-C34) in c-III differed by 3 to 10 nucleotides from their genuine haplotypes, which were determined to be heteroplasmic haplotypes.

Sequence size of haplotypes in groups B to D ranged from 774 to 810 bp. K2P distance between haplotypes of groups A and B ranged from 7.169 to 8.177% with a mean of 7.754 ± 0.973%, that between A and C ranged from 12.073 to 17.392% with a mean of 14.521 ± 1.151%, and that between A and D ranged from 17.472 to 23.880% with a mean of 21.042 ± 1.600%. Multiple stop codons were observed in a haplotype of group B, in five of eight haplotypes of group C, and all haplotypes of group D. Three haplotypes in group C had no stop codon but differed in four to 10 deduced amino acids from the genuine haplotypes. BLAST homology search revealed no identical sequence for haplotypes in groups B to D but indicated that the closest species were *P. japonicus* or *P. cygnus* with moderate similarity (83–89% homology). Therefore, all haplotypes of groups B to D (LC571565–LC571570, LC571572–LC571577, LC654683-LC654687) were determined to be NUMTs.

Among the 30–35 positive 12S clones examined per individual, sequences (772–806 bp) of 25–27 clones per individual (77 clones in total) were successfully determined. After alignment, primer sequences were trimmed, yielding 731–765 bp sequences. Thirteen clones of PJK1 were identical one another, as well as 12 of PJK2 and three of PJK3, and these were identical to PJK1-12Sdirect, PJK2-12Sdirect and PJK3-12Sdirect, respectively. These dominant haplotypes ranging from 761 to 762 bp in size were determined to be genuine 12S haplotypes of the individual, and representative sequences of these three genuine haplotypes were deposited in INSDC (LC605708‒LC605710). Nucleotide sequences of the remaining 49 clones were all different one another, in which 12 haplotypes were observed in PJK1, 23 in PJK2, and 14 in PJK3 (LC605711‒LC605748, OK429126–OK429131, LC654678-LC654682).

**Figure 2:**
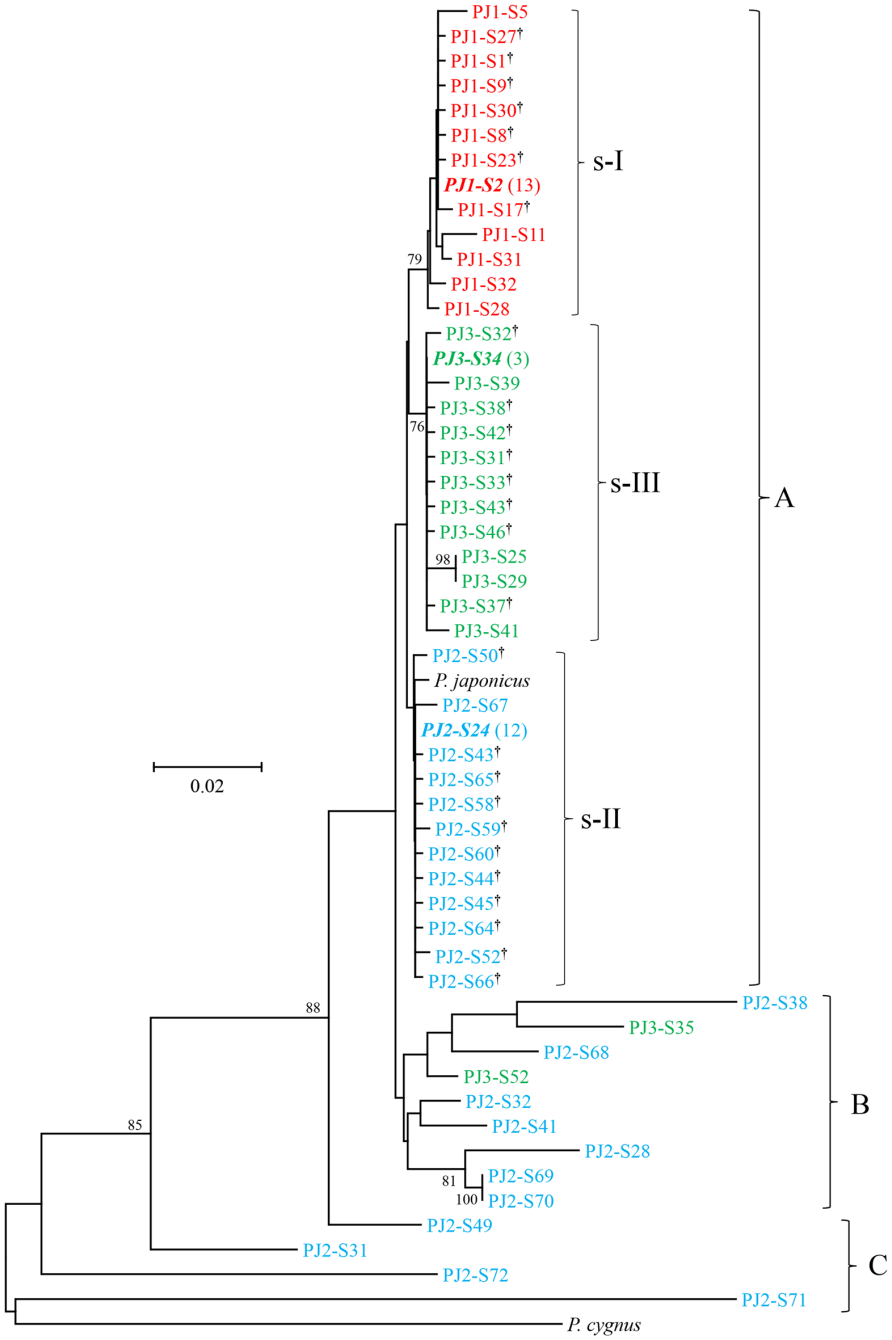
Neighbor-joining phylogenetic (NJ) tree showing relationships among 52 different haplotypes of clones of 12S rDNA (12S) or 12S-like sequences obtained from the Japanese spiny lobster (*Panulirus japonicus*), and 12S rDNA sequences of *P. japonicus* and *P. cygnus* derived from the GenBank database. Haplotypes detected from the same lobster individual share the same color. Genuine mtDNA haplotype is shown in bold italic and number of clones examined is shown in parenthesis. Haplotypes carrying dagger differ from corresponding genuine mtDNA haplotype by less than two nucleotides (including indel). The bootstrap values greater than 60% (out of 1000 replicates) are shown at the nodes.

Since incorporation of all eight *Panulirus* species sequences made sequence alignment ambiguous because of multiple indels, reference sequences of *P. japonicus* and of closely related *P. cygnus* were used for constructing phylogenetic tree (Fig. 2). Haplotypes detected from *P. japonicus* were segregated into three groups (designated by A to C). Sequence size of haplotypes in group A ranged from 760 to 762 bp. Three distinct clades (s-I to s-III) were observed in group A, in which 12 haplotypes each from PJK1, PJK2 and PJK3 were cohesively clustered together with their corresponding genuine haplotypes (bold italic). The numbers of variable nucleotide sites between haplotypes within s-I, s-II and s-III were 24, 17 and 16, respectively. Of these variable sites, transversion was observed at five, one and three sites, and indel was observed at one, zero and one sites, respectively. The mean K2P distances between different haplotypes within these clades ranged from 0.345 ± 0.081 to 0.519 ± 0.101%. The mean K2P distances between three clades ranged from 0.936 ± 0.275 to 1.371 ± 0.359%. Haplotypes differed by less than two substitutions (including indel) from the genuine haplotypes are marked with dagger. Five haplotypes in s-I clade and two haplotypes in s-III clade differed by three to six nucleotides from their genuine haplotypes, which were determined to be heteroplasmic copies.

Sequence size of haplotypes in group B varied from 731 to 762 bp. K2P distance between groups A and B ranged from 1.336 to 7.445% with a mean of 3.449 ± 0.398%, and those between a reference sequence of *P. japonicus* and groups A and B were 0.864 ± 0.236% and 3.189 ± 0.410%, respectively. Sequence size of haplotypes in group C varied from 744 to 765 bp. K2P distance between groups A and C ranged from 3.104 to 22.434% with a mean of 12.049 ± 0.901%, and those between a reference sequence of *P. japonicus* and group C ranged from 3.951 to 21.287% with a mean of 11.764 ± 0.901%. BLAST homology search indicated that the closest species for haplotypes in groups B and C was *P. japonicus* or *P. cygnus* with moderate to high similarity (84–98% homology). Therefore, all 13 haplotypes (LC605741‒LC605748, LC654678-LC654682) in groups B and C were determined to be NUMTs.

Among the 36–49 positive Dloop clones examined per individual, sequences (777–893 bp) of 26–38 clones per individual (92 clones in total) were successfully determined. After alignment, primer sequences were trimmed, yielding 736–853 bp sequences. Three clones (821 bp) of PJK1 were identical one another and determined to be genuine haplotype of this individual. Nine clones (813 bp) of PJK2 were identical to PJK2-Dloopdirect and determined to be genuine haplotype of this individual. Three clones (821 bp) of PJK3 were identical one another and determined to be genuine haplotype of this individual. Representative sequences of these three genuine haplotypes were deposited in INSDC (LC605750‒LC605752). Nucleotide sequences of the remaining 78 clones were all different one another, in which 25 haplotypes were observed in PJK1, 17 in PJK2, and 36 in PJK3 (LC605753‒LC605815, LC654419-LC654430, LC654675-LC654677).

**Figure 3:**
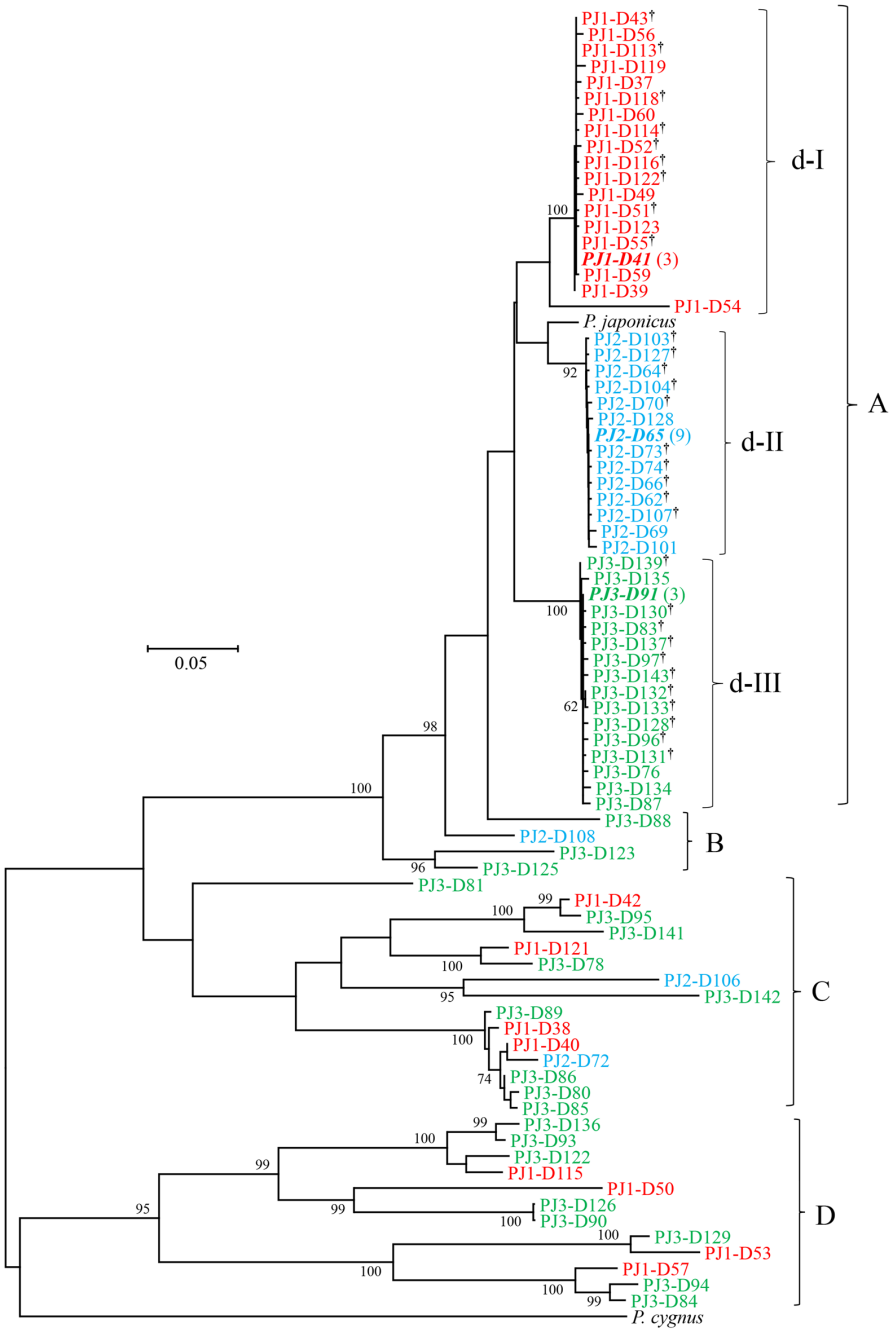
Neighbor-joining phylogenetic (NJ) tree showing relationships among 80 different haplotypes of control region (Dloop) or Dloop-like sequences obtained from the Japanese spiny lobster (*Panulirus japonicus*), and control region sequences of *P. japonicus* and *P. cygnus* derived from the GenBank database. Haplotypes detected from the same lobster individual share the same color. Genuine mtDNA haplotype is shown in bold italic and number of clones examined is shown in parenthesis. Haplotypes carrying dagger differ from corresponding genuine mtDNA haplotype by less than two nucleotides (including indel). The bootstrap values greater than 60% (out of 1000 replicates) are shown at the nodes.

Incorporation of all eight *Panulirus* species sequences made sequence alignment considerably unreliable because of multiple indels, reference sequences of *P. japonicus* and of closely related *P. cygnus* were used for constructing phylogenetic tree (Fig. 3). Haplotypes detected from *P. japonicus* were segregated into four groups (designated by A to D). Sequence size of haplotypes in group A ranged from 812 to 822 bp. Three distinct clades (d-I to d-III) were observed in group A, in which 17 haplotypes from PJK1, 13 from PJK2 and 15 from PJK3 were cohesively clustered together with their corresponding genuine haplotypes (bold italic). The numbers of variable nucleotide sites between haplotypes within d-I, d-II and d-III were 27, 61 and 28, respectively, of which indels were observed at five, two and four sites and transversion was observed at 0, six and six sites. The mean K2P distance between different haplotypes within these clades ranged from 0.340 ± 0.067 to 1.097 ± 0.139%. The mean K2P distance between these three clades ranged from 7.577 ± 0.951 to 8.770 ± 0.984%. Haplotypes differed by less than two substitutions (including indel) from the genuine haplotypes are marked with dagger. Eight haplotypes in d-I clade, three in d-II clade, and four in d-III clade differed by three to five nucleotides from the genuine haplotype were determined to be heteroplasmic copies.

Sequence size of haplotypes in groups B to D largely varied from 736 to 853 bp. K2P distances between group A and others ranged from 14.748 ± 1.030% (A vs B) to 61.619 ± 3.045% (A vs D), whereas that between haplotypes of group A and a reference sequence of *P. japonicus* was much smaller (6.333 ± 0.663%). BLAST homology search revealed no identical sequence for haplotypes in groups B to D and indicated that the closest species for haplotypes in groups B and C was *P. japonicus* with low to moderate similarity (74–88% homology). On the other hand, no significantly similar sequence was found for haplotypes in group D. Therefore, all 31 haplotypes (LC605788‒LC605815, LC654675-LC654677) in groups B to D were determined to be NUMTs.

### Impact of heteroplasmy and NUMTs for direct nucleotide sequencing

**Figure 4:**
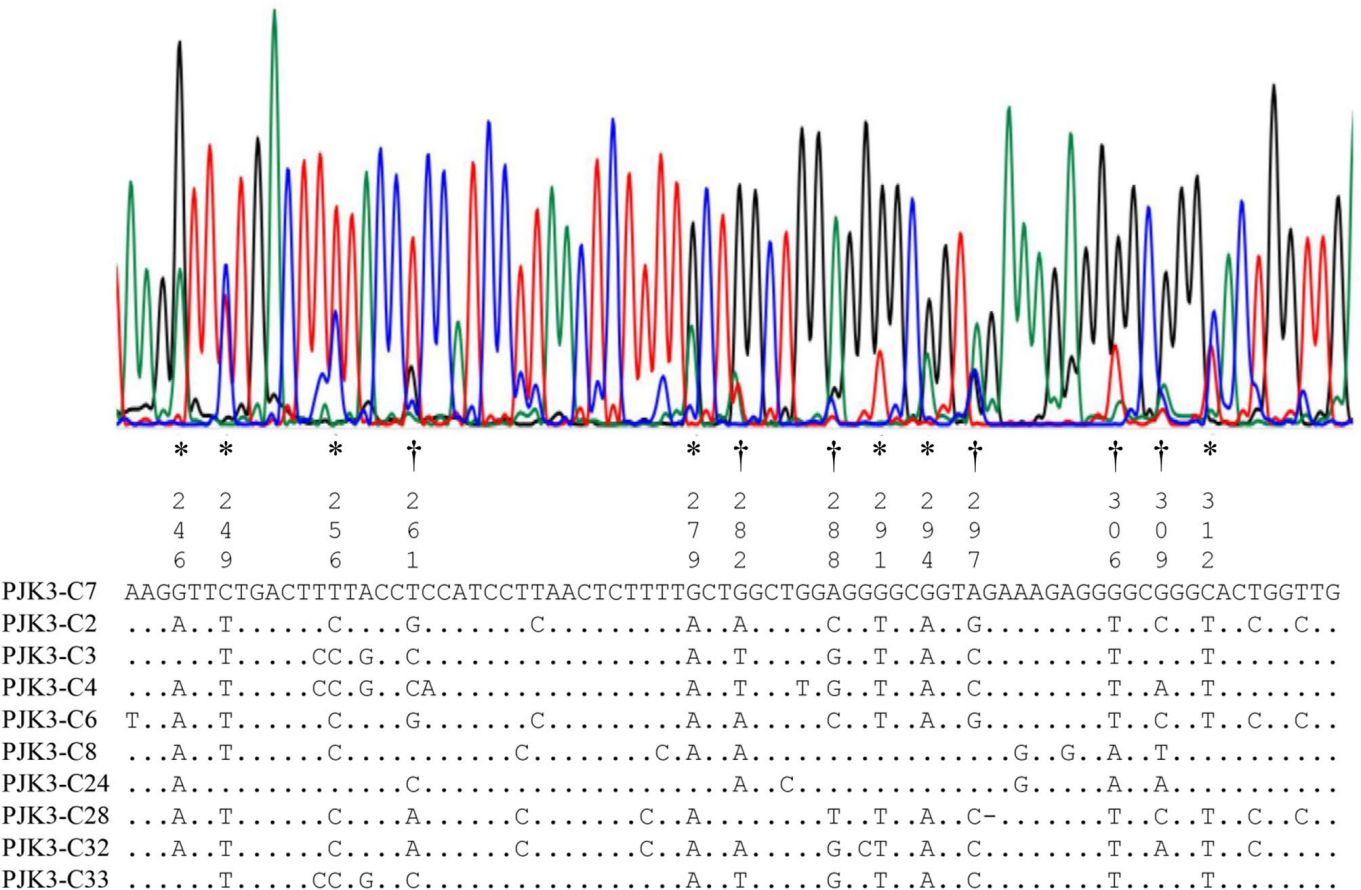
A part of electropherogram obtained by direct nucleotide sequencing for COI region of PJK3 (top), and corresponding sequences from genuine haplotype (PJK3-C7) and nine NUMT haplotypes (see Fig. 1) are aligned (bottom). Apparent double (asterisk) and triple (dagger) peaks are observed at seven and five sites, respectively, which are comprised of signals from genuine and NUMT haplotypes.

Partial electropherogram obtained by direct nucleotide sequencing for COI amplicon of PJK3 is shown in Fig. 4 (top). Peak signals of this electropherogram are readable, but there are a number of sites where two (asterisk) or three (dagger) signals overlap. Alignment of a genuin haplotype (PJK-C7) and nine NUMTs sequences, corresponding to this partial electropherogram, is shown in Fig. 4 (bottom). At the sites where plural peaks overlap, different NUMT haplotypes were observed to share the same nucleotide different from the PJK3-direct. Heteroplasmic copies in COI determined in this study may have little negative impact on direct nucleotide sequencing, since nucleotides different from the genuine haplotypes were all unique to each heteroplasmic haplotype. Thus, the plural peaks at a site were composed of signals from genuine plus NUMT haplotypes, and the intensity of each peak was positively related to the copy numbers of these haplotypes. Frequent failure to obtain readable electropherograms in 12S and Dloop regions by direct sequencing may be due to extensive indels observed in the NUMT haplotypes.

## Discussion

### Heteroplasmy

In order to determine genuine and heteroplasmic mtDNA and NUMT sequences, we primarily relied on phylogenetic analysis with supplementary information of synonymous, nonsynonymous and nonsense mutations in COI and COI-like sequences, nucleotide sequence divergence, and extent of indels among sequences. Heteroplasmy in mtDNA has been ubiquitously observed in many eukaryotes^3,4^, but its abundance is expected to be low, specifically in coding regions. However, extensive heteroplasmy has been revealed even in coding regions of several crustacean species^5,6,15–17^. Almost all sequences in group A were cohesively clustered together with their corresponding genuine haplotypes, clearly negating the possibility that these variable sequences are derived by cross-contamination. Although we determined some variable sequences to be heteroplasmic copies based on a tentative criterion, it is actually difficult to distinguish heteroplasmic copies from variants caused by amplification error without cDNA analysis. Among 108 COI sequences of Japanese spiny lobster analyzed, nonsynonymous substitution was observed only in two individuals^9,10^. Chan et al.^10^ reported partial COI nucleotide sequences (657 bp) in 35 Japanese spiny lobsters collected around Taiwan, in which 47 of 48 variable sites observed among individuals were at the 3rd position of a codon, and nonsynonymous substitution was observed only at one site of an individual. These values are considerably lower than those observed between variant sequences and the genuine haplotypes of group A found in the present study. Rates of synonymous substitution were higher than those of nonsynonymous substitution in mtDNA functional regions of fish and mammals^18,19^. Specifically in COI gene, rate of synonymous substitutions was 62 times higher than that of nonsynonymous substitutions^20^. Therefore, it is likely that not all of the heteroplasmic copies are expressed, consistent with the results of other studies, which reported only one cDNA sequence from a single individual of snapping shrimp and blue crab was observed^5,15^. Williams et al.^15^ suggested the possibility that heteroplasmic copies are not expressing, postulating mechanisms to achieve their silencing.

### NUMTs

Bensasson et al.^21^ stated in their review that abundance of NUMT varied among taxa, to the extent that very few NUMT have been reported in fish and crustaceans. However, more recent investigations as well as the present study contradict empirical interpretations that NUMTs are virtually nonexistent in fishes and uncommon in crustaceans^5–7,15,17,22–26^. Therefore, it is likely that during analysis of the coding regions of mtDNA, sequences that could not be translated to deduced amino acid sequences might have been unreported or discarded without being registered.

As in the case of heteroplasmy, we need to rule out the possibility of cross-contamination also for NUMTs detected. BLAST homology searches revealed no identical sequence in database for the NUMTs but indicated that all NUMTs except for those in group D of Dloop were highly to moderately homologous to the corresponding mtDNA regions of *P. japonicus* or *P. cygnus*. There have been 22 species described in the genus *Panulirus*^27^, and COI, 12S and Dloop data are available in the database for 21, 16 and 13 species, respectively. All spiny lobsters are large, well known and economically important benthic animal. Therefore, it is unlikely that we have detected contaminant sequences of unknown or cryptic lobster species.

Our phylogenetic analysis revealed considerable diversity of the NUMTs detected in the Japanese spiny lobster, consistent with the implication that integration of mtDNA into the nuclear genome is a continuous and dynamic process^28^. Some NUMT haplotypes are shown to be closely clustered (Figs. 1, 2 and 3), suggesting these to be alleles in a locus. As mentioned earlier, however, the number of alleles may be overestimated due to *Taq* polymerase error.

Our phylogenetic analysis also suggested that NUMTs of group D of COI and group D of Dloop were integrated into nuclear genome at a time similar to speciation events and groups B and C after speciation events. All NUMTs detected in the present study may be relatively young, since sequence divergences between these NUMTs and the genuine haplotypes are comparable with, or smaller than, those between good species of the genus^12,29^. However, the number and extent of NUMTs detected in the present study must be a considerable underestimate of the true number, since we observed a number of clones with inserts much shorter than that of genuine haplotype but excluded them from analysis. Furthermore, older NUMTs may have become shortened, fragmented, degenerated, and/or accumulated substantial nucleotide substitutions^1,2,5,30–32^, resulting in no longer having a complementary sequence for the primers used in this study.

### Effects of heteroplasmy and NUMTs

One might expect that the presence of heteroplasmic copies and/or NUMTs may be insignificant because of the copy number smaller than genuine mtDNA, and that sequences obtained by direct sequencing of PCR amplicons from the mtDNA are representatives of the most common haplotypes within an individual. Indeed, we could obtain analyzable electropherograms in COI of all three individuals by direct sequencing despite the presence of heteroplasmic and NUMT haplotypes. However, we also noted that heteroplasmic haplotypes and NUMTs co-amplified with genuine haplotypes may have a slight to significant impact on the quality of electropherogram obtained by direct nucleotide sequencing. Although nearly half or more of clones examined in each lobster individual were variant haplotypes similar to the genuine sequences, a small number of point mutations unique to each haplotype had little effect on the electropherogram. In contrast, multiple NUMTs that shared the same nucleotide substitution and differed from the genuine haplotype, contributed to double or triple peaks (Fig. 4). Direct nucleotide sequencing of PCR amplicons of mtDNA control region from blood sample (depauperate in mtDNA) in bird showed ambiguous (= double peak) sites, but those from other tissues did not, indicating a substantial contamination from NUMTs^33^. Multiple indels between NUMTs and genuine haplotypes would have a significant impact on the resultant electropherograms, which likely happened in direct sequencing for 12S and Dloop regions in the present study. Furthermore, unnoticed shorter NUMTs must also contribute to the negative impact on the electropherogram. Thus, NUMTs not only make sequence analysis difficult, but sometimes the sequences may be erroneously adopted as genuine mtDNA sequences^2^. To circumvent the effect of NUMTs, the use of mtDNA-rich tissue, mtDNA enrichment, dilution of template DNA, protein-coding regions, long PCR, or cDNA analysis has been recommended^2,5,34^. Further, advance information on the abundance of NUMTs and heteroplasmy in any species of interest would be highly advantageous to address their negative impact. When one encounters consistent problems with obtaining good electropherograms in PCR-amplified mtDNA, it would be recommended to design PCR primers that avoid annealing to NUMTs.

Advances in detection technologies, such as next-generation sequencing and bioinformatics have made it possible to generate and analyze a huge number of sequences and identify even minor sequences that have been difficult to detect by the Sanger method and small-scale clone libraries. Thus, a database of NUMTs and heteroplasmic copies across species would be important not only for quality control of nucleotide sequences obtained but also for evolutionary phylogenetic inference.

## Conclusions

Mitochondrial DNA (mtDNA) has been widely used in molecular phylogenetics, population genetics, and DNA barcoding, and direct nucleotide sequencing for PCR amplicons of mtDNA has been a conventional tool to detect sequence variation within and between species. Although reports on nuclear mitochondrial pseudogene referred to as “NUMT” and heteroplasmy are increasing in a wide range of eukaryotes, little attention has been paid to their impacts on the direct nucleotide sequencing. In this study, we detected NUMTs and heteroplasmy in the Japanese spiny lobster and observed that specifically the NUMTs had a negative impact to obtain good electropherograms. Unnoticed incorporation of heteroplasmic copies and NUMTs may lead to overestimation of population diversity and the number of species or individuals.

## Materials and methods

Three individuals (designated by PJK1 to PJK3) of the Japanese spiny lobster (*Panulirus japonicus*) used in this study were caught in Chiba Prefecture, Japan in 1996, and the walking legs were fixed in 80% ethanol and kept in the laboratory of the Fisheries Resources Institute, Japan Fisheries Research and Education Agency. Crude DNA was extracted from the muscle using a DNA extraction kit (QuickGene DNA tissue kit, DT-S, KURABO). To amplify three segments of mitochondrial DNA (COI, 12S, and Dloop), we designed semi-species-specific primers using whole mtDNA reference sequences of the following eight *Panulirus* species in the GenBank database: *P. argus* (NC_039671), *P. cygnus* (NC_028024), *P. homarus* (JN542716), *P. japonicus* (NC_004251), *P. ornatus* (GQ223286), *P. polyphagus* (MK503959), *P. stimpsoni* (GQ292768), and *P. versicolor* (NC_028627). The primer sequences to amplify COI, 12S and Dloop regions are as follows: PanJCOIF: 5’-ACGCAACGATGATTTTTCTCTAC-3’ and PanJCOIR: 5’-ACAGCAATAATTATGGTTGCCG-3’ (for COI); PanJ12SF: 5’-TTAATGAAAGCGACGGGCAA-3’ and PanJ12SR: 5’-CCTATAGTTTGATTCTTGCTA-3’ (for 12S); PanJ12SF2: 5’-TAGCAAGAATCAAACTATAG-3’ and PanJtRNAR: 5’-ACATTACTTGCTCTATCAAA-3’ (for Dloop). According to the reference sequence, amplified fragment size of the Japanese spiny lobster using these primer pairs were expected to be 935, 802, and 863 bp for COI, 12S and Dloop regions, respectively.

PCR amplification was performed in 12 μL reaction mixture containing 1 μL of template DNA (1–10 ng/μL), 1.2 μL of 10 × reaction buffer, 1.2 μL of dNTP (2.5 mM each), 0.7 μL of each primer (10 μM), 0.3 μL of EX Taq HS polymerase (5 units) (Takara Bio, Inc.), and 7.6 μL of distilled water. The reaction mixtures were preheated at 94 °C for 5 min, followed by 35 amplification cycles (denaturation at 94 °C for 0.5 min, annealing at 58 °C for 0.5 min and extension at 72 °C for 1 min), with a final extension at 72 °C for 7 min. The PCR products were electrophoresed on a 1.5% agarose gel to confirm amplification and treated with ExoSAP-IT (GE Healthcare) to remove PCR primers. Direct nucleotide sequencing was performed using a BigDye Terminator Ver3.1 kit (Applied Biosystems) with forward and reverse PCR primers. Sequencing was conducted on an ABI3730XL automatic sequencer (Applied Biosystems). When a double peak was observed, the stronger signal was adopted. The PCR products were cloned using a DynaExpress TA PCR Cloning Kit (BioDynamics Laboratory Inc.). Colony-direct PCR was performed using M13 primers or PCR primers with the reaction protocol described above, followed by agarose gel electrophoresis to confirm the size of the fragments inserted. No further experiment was performed on clones with apparently different insert size from the target regions. The amplicons were treated with ExoSAP-IT and subjected to nucleotide sequencing with M13 primers or PCR primers. Electropherograms obtained from clone libraries were carefully checked, and those with ambiguous peaks were discarded as they likely resulted from picking multiple colonies. All nucleotide sequences determined were subjected to BLAST homology searches^35^ in GenBank to find identical or similar sequences. Translation to deduced amino acid sequences for COI were performed using GENETYX ver. 12 (GENETYX Co., Tokyo). Nucleotide sequence alignment was performed using the ClustalW algorithm^36^ in MEGA 6^37^ followed by manual editing. Calculation of K2P distance between sequences under pairwise deletion option and construction of neighbor-joining (NJ) phylogenetic tree were performed using MEGA 6^37^.
