# Author Correction: Detection of heteroplasmy and nuclear mitochondrial pseudogenes in the Japanese spiny lobster *Panulirus japonicus*

**DOI:** 10.1038/s41598-022-08715-x

**Published:** 2022-03-15

**Authors:** Seinen Chow, Takashi Yanagimoto, Haruko Takeyama

**Affiliations:** 1 Research Organization for Nano and Life Innovation, Waseda University, 513 Wasedatsurumaki-cho, Shinjuku-ku, Tokyo 162-0041 Japan; 2 Fisheries Resources Institute, Japan Fisheries Research and Education Agency, Fukuura 2-12-4, Yokohama, Kanagawa 236-8648 Japan; 3 Department of Life Science and Medical Bioscience, Waseda University, 2-2 Wakamatsu cho, Shinjuku, Tokyo 162-8480 Japan; 4 Computational Bio Big-Data Open Innovation Laboratory, AIST-Waseda University, 3-4-1 Okubo, Shinjuku-ku, Tokyo 169-0072 Japan

Correction to: *Scientific Reports* 10.1038/s41598-021-01346-8, published online 05 November 2021

The original version of this Article omitted an affiliation for Seinen Chow. The correct affiliations are listed below.

Research Organization for Nano and Life Innovation, Waseda University, 513 Wasedatsurumaki‑cho, Shinjuku‑ku, Tokyo 162–0041, Japan.

Fisheries Resources Institute, Japan Fisheries Research and Education Agency, Fukuura 2‑12‑4, Yokohama, Kanagawa 236‑8648, Japan.

The original Article has been corrected.

